# Multicenter randomized trial of a digital therapeutic game for executive function in children with ADHD

**DOI:** 10.1038/s41746-026-02721-3

**Published:** 2026-05-27

**Authors:** Sojung Lee, You Bin Lim, Wooseok Choi, Sohyun Park, Bo-Ram Han, Seonyoung Lee, Suhjung Lee, You Kyung Lim, Seungbee Lim, Eunice Jiun Chung, Dongwon Kang, Inyoung Son, Jungsang Min, Johanna Inhyang Kim, Bung-Nyun Kim

**Affiliations:** 1 eMotiv Co. Ltd, Seoul, Republic of Korea; 2 Division of Child and Adolescent Psychiatry, Department of Psychiatry, Seoul National University College of Medicine, Seoul, Republic of Korea; 3 Clinical Team, AP Korea, Seoul, Republic of Korea; 4 Department of Psychiatry, College of Medicine, Hanyang University, Seoul, Republic of Korea

**Keywords:** Diseases, Health care, Medical research, Neurology, Neuroscience, Psychology, Psychology

## Abstract

Significant executive function deficits mark attention-deficit/hyperactivity disorder (ADHD). Digital therapeutics (DTx) targeting cognitive control offer promising non-pharmacological options but require rigorous validation. We evaluated the efficacy and safety of STAR RUCKUS, an adaptive multitasking DTx designed to strengthen cognitive control in children with ADHD. In this multicenter, randomized, single-blind, sham-controlled trial, 122 children aged 6–12 years with DSM-5/ICD-10–diagnosed ADHD were assigned 1:1 to STAR RUCKUS or a visually matched driving-only sham intervention and completed five daily sessions for 4 weeks. The primary outcome was the change in clinician-rated Korean ADHD rating scale (K-ARS) scores. Of 122 randomized children, 114 completed the trial. At Week 4, STAR RUCKUS produced greater symptom reduction than sham (−7.50 vs. −4.00; *p* = 0.009), with improvements in inattention (*p* = 0.024) and hyperactivity/impulsivity (*p* = 0.015). These findings suggest statistically significant improvements of small-to-moderate magnitude (*ηp*² = 0.05) in clinician-rated ADHD symptoms, along with favorable adherence and tolerability. Trial registration: ClinicalTrials.gov (NCT07173439).

## Introduction

Attention-deficit/hyperactivity disorder (ADHD) is a neurodevelopmental disorder with prevalence rates ranging from approximately 5–10% in children^1,2^. Its characteristics include persistent symptoms of inattention, impulsivity, and hyperactivity that result in significant impairments in academic achievement, family functioning, and social interactions^3^. Currently, first-line treatments for ADHD typically include pharmacological interventions, particularly stimulant medications, which have shown short-term effectiveness in symptom improvement^4–7^. However, limitations such as side effects, stigma toward ADHD medication, and challenges in treatment accessibility pose significant barriers to long-term ADHD management^5,8,9^. Recently, digital therapeutics (DTx) have emerged as an alternative treatment approach that could potentially address some of these limitations^10^. Multitasking-based cognitive training delivered as DTx leverages mobile technology to maximize accessibility, personalization, and safety^11–13^. Several DTx interventions specifically target executive function (EF), a key neurocognitive function closely linked to ADHD symptoms^10,14^.

Scholars, given this focus on EF, scholars identified impaired EF as a core underlying deficit in ADHD^14–16^. EF encompasses a set of cognitive processes, including attentional selection and working memory, that enable goal-directed behavior^17–20^. Within this framework, cognitive control regulates attention and behavior, particularly under distraction^19,20^. Neuroimaging studies link these cognitive control deficits to abnormal functional activity and connectivity in frontoparietal networks, especially in the dorsolateral prefrontal cortex (DLPFC) and anterior cingulate cortex (ACC)^20,21^. Researchers also identify frontal midline theta (FMT)^22^ as a neural marker of cognitive control. Multitasking-based DTx, which aligns conceptually with FMT-related cognitive control processes, shows promise in improving attentional performance and clinical ADHD symptoms in pediatric populations^23^. These findings provide a theoretical basis for multitasking interventions grounded in cognitive control theories, which the present trial evaluates through clinical and behavioral outcomes.

Despite this strong theoretical and empirical support, multitasking-based DTx trials exhibit several methodological limitations. For example, one prior study^24^ combined a go/no-go task with a driving simulation, while another^25^ integrated touchscreen-based multitasking and augmented reality-based physical exercise to enhance attention, working memory, and inhibitory control. Both studies reported improvements in cognitive inhibition and EF. However, two critical methodological issues limit the clinical validity and credibility of these studies.

First, one study^26^ argued that a prior trial^24^ demonstrated improvements primarily on computerized attention tests (near transfer) without clear evidence of far transfer to clinician-rated ADHD symptoms. Another investigation^25^ relied exclusively on parent-rated scales, which are vulnerable to reporter bias and single-context limitations, resulting in modest agreement with clinician or teacher ratings^27,28^. Second, these trials employed inadequately designed sham controls. The trial^24^ used a puzzle-solving control condition that differed substantially from the active multitasking intervention, and the later study^25^ relied on a wait-list control, which does not sufficiently account for placebo or expectancy effects.

Therefore, rigorous sham controls that closely match active interventions in user experience, engagement, and blinding are essential to form a critical methodological pillar that can ensure the clinical credibility of multitasking-based DTx research^11^. We selected an adaptive N-back task because it preferentially engages proactive cognitive control functions, such as sustained working memory maintenance and proactive goal updating, rather than simple response inhibition. Evidence suggests that these proactive mechanisms correlate closely with core ADHD symptoms, including inattention and impulsivity^21,29^.

We aimed to overcome these methodological limitations and establish the clinical efficacy of multitasking-based DTx by implementing three rigorously designed refinements. First, we used clinician-rated symptom measures as primary outcomes to overcome inherent biases in surrogate cognitive tests and parent ratings. Second, we implemented a rigorously matched sham control (driving simulation) to replicate the interface, user engagement, session duration, and blinding of the active intervention, effectively controlling for placebo and expectancy effects. Third, we selected an adaptive N-back task as the primary cognitive control component instead of simpler response-inhibition tasks because it preferentially engages proactive cognitive control functions, such as sustained working memory maintenance and proactive goal updating.

These proactive cognitive mechanisms are thought to play an important role in the core symptoms of ADHD, including inattention and impulsivity, and have been associated with prefrontal–parietal cognitive control circuits implicated in ADHD^20,21,29–31^. Preliminary results from our pilot study, an open-label trial with 22 children and no control group, showed significant reductions in inattentive and hyperactive-impulsive ADHD symptoms following a four-week multitasking intervention, independent of medication status^32^. Collectively, these refinements enable this study to evaluate the clinical efficacy and practical value of multitasking-based DTx for treating ADHD.

## Results

Between April 15, 2024, and March 6, 2025, we assessed 156 children for eligibility and excluded 34 who did not meet the inclusion criteria. We then randomly assigned the remaining 122 eligible participants in a 1:1 ratio to the STAR RUCKUS group (*n* = 62) or the sham control group (*n* = 60).

**Fig. 1:**
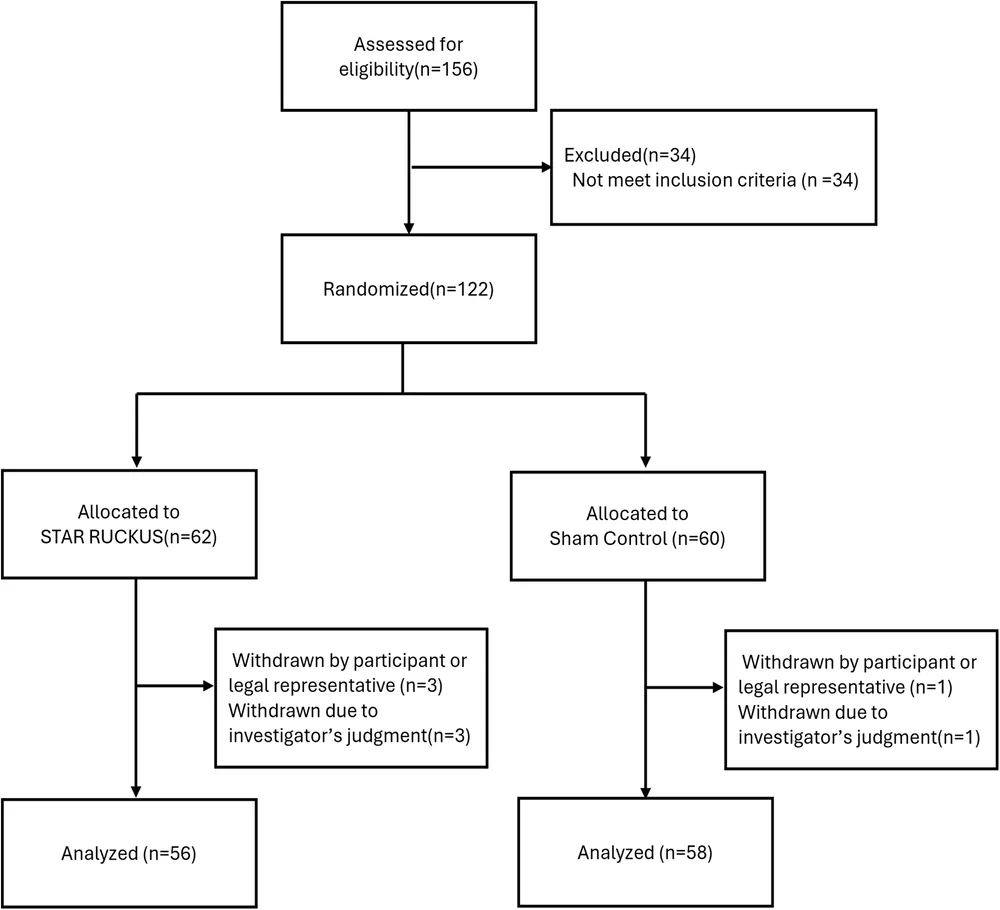
Trial profile. Flow diagram showing participant screening, randomization, allocation, and analysis in the multicenter randomized trial.

In the STAR RUCKUS group, six participants discontinued before the post-intervention assessment: three withdrew consent (personally or through their legal guardians), and the investigator exercised his discretion to withdraw the other three. Therefore, the intervention group included 56 participants in the primary analysis. In the sham control group, two participants discontinued (one due to voluntary withdrawal and one due to the investigator’s decision), yielding 58 participants for the final analysis. Figure 1 illustrates the flow of participants through each phase of the trial.

The baseline demographic and clinical characteristics were well balanced between the STAR RUCKUS (*n* = 56) and sham control (*n* = 58) groups. The mean age was 8.61 years (SD = 1.74) in the intervention group and 8.45 years (SD = 1.79) in the control group. The proportion of male participants was comparable between groups (73.2% vs. 72.4%).

Baseline medication status was similar, with 46.4% and 43.1% of participants on stable pharmacological treatment in the intervention and control groups, respectively. ADHD symptom severity at baseline, assessed using the clinician-administered K-ARS, did not differ significantly between groups in total scores (28.7 ± 6.72 vs. 28.9 ± 6.41), inattentive symptoms (16.6 ± 3.44 vs. 16.2 ± 3.20), or hyperactive/impulsive symptoms (12.1 ± 4.52 vs. 12.7 ± 5.02). Table 1 summarizes the participants’ baseline characteristics, supporting successful randomization and baseline comparability (all *p*-values > 0.05).

**Table 1:** Baseline demographic and clinical characteristics by group

	STAR RUCKUS (*N* = 56)		Sham Control (*N* = 58)		*P* Value
	M	SD	M	SD	
Age	8.61	1.74	8.45	1.79	0.57
Sex					0.92
Male, (*N*, %)	41	73.2	42	72.41	
Female, (*N*, %)	15	26.79	16	27.59	
Medication					0.72
Yes, (*N*, %)	26	46.4	25	43.1	
No, (*N*, %)	30	53.5	33	56.0	
K-ARS					
Total	28.7	6.72	28.9	6.41	0.87
Inattention	16.6	3.44	16.2	3.20	0.54
Hyperactivity-impulsivity	12.1	4.52	12.7	5.02	0.51

*P* values were calculated using independent t-tests for continuous variables and chi-square tests for categorical variables.

*K-ARS* Korean ADHD rating scale.

At Week 4, mean K-ARS total scores decreased by 7.50 (SD = 7.73) points in the STAR RUCKUS group and 3.92 (SD = 8.27) points in the sham control group. The adjusted between-group effect was significant (*p* = 0.009, *ηp*² = 0.05; Table 2). In post hoc analyses, the Inattention subscale showed a significantly greater decrease in the intervention group than in the control group (*p* = 0.023, *ηp*² = 0.04), with mean pre–post changes of −4.02 and −2.33 points, respectively. The Hyperactivity/Impulsivity subscale also favored the intervention group (*p* = 0.015, *ηp*² = 0.04), with decreases of −3.49 and −1.67 points in the intervention and control groups, respectively.

**Table 2:** Within- and between-group changes in outcome measures (K-ARS Total and Subscales)

	STAR RUCKUS (*N* = 56)				Sham control (*N* = 58)				Between-group	
	Pre (M, SD)	Post (M, SD)	*P*	*d*	Pre (M, SD)	Post (M, SD)	*P*	*d*	*p*	*ηp*²
K-ARS										
Total	28.70 (6.72)	21.20 (8.40)	<0.001	0.97	28.90 (6.41)	24.98 (7.55)	<0.001	0.48	**0.009**	0.05
Inattention	16.57 (3.44)	12.55 (4.72)	<0.001	0.86	16.19 (3.20)	13.86 (4.16)	<0.001	0.57	**0.024**	0.04
Hyperactivity-impulsivity	12.13 (4.52)	8.64 (4.97)	<0.001	0.85	12.67 (5.02)	11.00 (4.62)	0.009	0.35	**0.015**	0.04
Clinical global impression-improvement		3.11 (0.82)				3.38 (0.64)			**0.033**	0.03

*P*-values were derived from analysis of covariance (ANCOVA), adjusting for baseline K-ARS score, sex, and medication status as covariates. *d* indicates Cohen’s *d*. *ηp*² indicates partial eta squared.

*K-ARS* Korean ADHD rating scale.

**Fig. 2:**
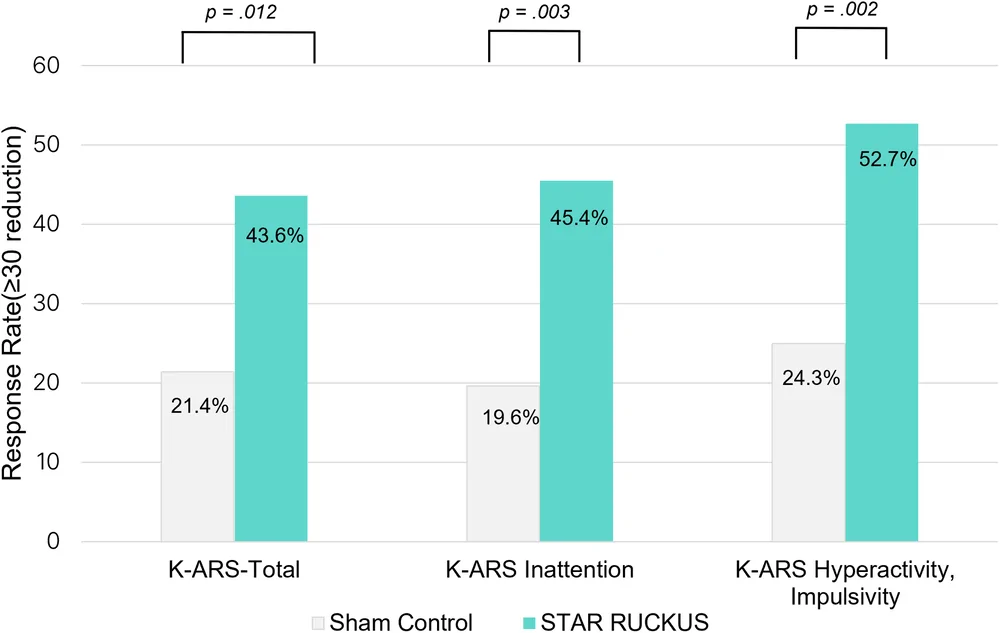
Clinical response rates following STAR RUCKUS intervention in children with ADHD. Clinical response was defined as a ≥30% reduction in Korean ADHD rating scale (K-ARS) scores. Gray bars indicate the sham control group, and teal bars indicate the STAR RUCKUS intervention group. *P* values were calculated using chi-square tests.

Consistent with these effects, a higher proportion of participants in the intervention group met the ≥30% K-ARS response criterion. Response rates were 43.6% compared with 21.4% for the total score (*p* = 0.012, Cramer’s *V* = 0.25), 45.4% compared with 19.6% for inattention (*p* = 0.003, Cramer’s *V* = 0.27), and 52.7% compared with 24.3% for hyperactivity/impulsivity (*p* = 0.002, Cramer’s *V* = 0.30; Fig. 2).

Among predefined secondary efficacy outcomes, the STAR RUCKUS group showed nominally greater improvement in two neurocognitive domains: attentional sensitivity, as assessed by the ADS Visual Sensitivity Index (*d*′), and cognitive inhibition, as assessed by the Stroop Color–Word subtest. The intervention group also showed significantly greater improvement in ADS visual sensitivity (*d*′) (*p* = 0.021, *ηp*² = 0.04). Stroop Color–Word subtest performance improved significantly in the intervention group compared with the control group (*p* = 0.025, *ηp*² = 0.04). However, these neurocognitive effects did not remain statistically significant after Benjamini–Hochberg FDR correction and should therefore be interpreted as exploratory (Supplementary Table 2).

In contrast, Clinician-rated global symptom improvement measured by the CGI-I scale was significantly greater in the STAR RUCKUS group (3.11 ± 0.82) than in the sham control group (3.38 ± 0.64, *p* = 0.033, *ηp*² = 0.03), indicating greater overall clinical improvement in the participants who received the active intervention.

We conducted exploratory post hoc subgroup analyses based on concurrent medication status (medicated vs. non-medicated). These analyses included participants aged ≥8 years and excluded those with training adherence below the 7th percentile (approximately 1.5 SD below the mean).

**Fig. 3:**
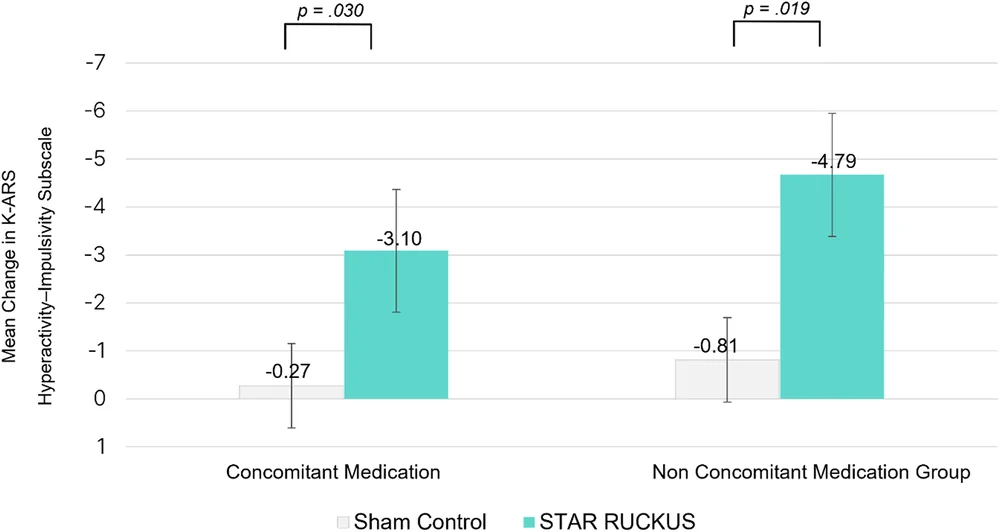
Mean change in K-ARS hyperactivity impulsivity subscale following STAR RUCKUS intervention. Mean change in the Korean ADHD Rating Scale (K-ARS) hyperactivity impulsivity subscale in children receiving the STAR RUCKUS intervention compared with the sham control group. Gray bars indicate the sham control group, and teal bars indicate the STAR RUCKUS intervention group. Error bars indicate the standard error of the mean. *P* values were calculated using Mann–Whitney U tests.

For the K-ARS Hyperactivity–Impulsivity subscale, greater reductions were observed in the STAR RUCKUS group compared with the sham control group in both the medicated subgroup (−3.10 ± 4.39 vs. −0.27 ± 4.54; *p* = 0.030; *r* = −0.41) and the non-medicated subgroup (−4.79 ± 4.87 vs. −0.81 ± 4.19; *p* = 0.019; *r* = −0.43; Fig. 3).

For K-ARS total scores, no statistically significant between-group differences were observed in either the medicated subgroup (−7.05 ± 8.03 vs. −2.20 ± 7.54; *p* = 0.091; *r* = −0.33) or the non-medicated subgroup (−9.50 ± 9.20 vs. −2.57 ± 7.15; *p* = 0.066; *r* = −0.35).

Similarly, for the Inattention subscale, no statistically significant between-group differences were observed in the medicated subgroup (−3.95 ± 4.60 vs. −1.93 ± 3.79; *p* = 0.139; *r* = −0.29) or the non-medicated subgroup (−4.71 ± 5.53 vs. −1.76 ± 3.39; *p* = 0.090; *r* = −0.32).

We conducted sensitivity analyses to examine the robustness of the primary efficacy outcome. These included comparisons between LOCF and complete-case analyses, as well as between the full analysis set and per-protocol set. We also applied robust regression to account for potential outliers and violations of the model assumptions. The direction and statistical significance of the treatment effect remained consistent across all approaches, indicating robust results. We observed differences between groups in parental characteristics; therefore, we did not conduct further sensitivity analyses for these variables.

Mean adherence rates, calculated as the number of completed sessions (with a maximum of 100), were 90.8 (SD = 14.7) and 90.7 (SD = 11.7) in the STAR RUCKUS and sham control groups, respectively, indicating high and comparable engagement across groups (*p* = 0.992). When expressed using the raw number of completed sessions (without imposing the 100-session cap), participants completed an average of 102.46 sessions (SD = 26.12) in the STAR RUCKUS group and 103.76 sessions (SD = 26.05) in the sham control group, with no significant difference between groups (*p* = .948). Post-intervention, participants rated their overall satisfaction with the intervention at 6.38 (SD = 2.04) on a 10-point visual analog scale, suggesting moderate-to-high perceived acceptability. None of the participants initiated or discontinued ADHD medication, and there were no changes in medication dosage in either group during the intervention period. Two participants (3.57%) in the STAR RUCKUS group reported a device-related adverse event (somnolence), whereas none occurred in the sham control group. All reported events were mild in severity, transient in duration, and did not result in study discontinuation. Neither group reported serious adverse events during the study.

## Discussion

This randomized sham-controlled clinical trial suggested that STAR RUCKUS, a multitasking-based DTx, improves pediatric ADHD symptoms over four weeks. Compared with the visually matched sham control group, the intervention group demonstrated significantly greater reductions in inattentive and hyperactive–impulsive symptoms on the K-ARS, as rated by psychiatrists who remained blinded to treatment allocation. The intervention group also achieved higher treatment response rates.

Objective neuropsychological assessments showed improvements in attentional sensitivity (d′) and inhibitory control; however, these effects did not remain statistically significant after correction for multiple comparisons and therefore remain exploratory. Stratified analyses by medication status indicated a similar direction of symptom improvement across medicated and non-medicated subgroups, although these findings also remain exploratory.

The present study strengthened internal validity and minimized expectancy-related confounds by using a visually matched sham control that included the same driving task but excluded the multitasking component, along with blinded clinician-rated assessments. Prior digital therapeutic research for ADHD produced variable findings regarding far-transfer effects on clinician-rated outcomes^24,25,33,34^.

Within this broader literature, the intervention’s multitasking configuration targets multiple cognitive control processes, including proactive and reactive regulation, which researchers have implicated in ADHD^14,19,29^. Meta-analyses highlight the potential importance of engaging multiple EF domains in ADHD interventions^35,36^. Complementing this perspective, neurocognitive research grounded in the dual control model describes disruptions in proactive and reactive control processes among children with ADHD^29^. Building on this theoretical and empirical foundation, we designed the multitasking configuration to engage these regulatory processes.

The intervention combined an adaptive N-back task—designed to engage sustained working memory updating and anticipatory regulation— with a concurrent driving simulation that required visuomotor integration and inhibitory control. The concurrent improvements observed in clinician-rated symptoms and selected executive-function measures align with the theoretical premise that engaging multiple domains of cognitive control may influence behavioral symptom expression in ADHD^35,36^. However, because the cognitive findings did not remain statistically robust after correction for multiple comparisons and we did not directly assess neural activity, mechanistic interpretations remain tentative.

Taken together, the primary clinician-rated outcomes support consideration of STAR RUCKUS within multimodal ADHD care. As a non-pharmacological digital intervention, it complements treatment for children who experience challenges with medication adherence or tolerability^37–40^. However, the subgroup findings remain exploratory, and the study lacked sufficient power to evaluate interaction effects. Future research should clarify the role of combined digital and pharmacological interventions.

Despite these promising findings, several limitations warrant consideration. First, the relatively short four-week intervention period limits conclusions regarding the long-term durability of treatment effects. Although participants showed symptom improvements during the active intervention phase, it remains uncertain whether these effects would persist, diminish, or require sustained engagement beyond the study period. Future longitudinal studies with extended follow-up periods should clarify the long-term stability of these gains^41,42^.

Second, the study does not provide mechanistic evidence linking the observed clinical effects to specific neural processes. Although we grounded the intervention conceptually in neurocognitive models of cognitive control, the findings do not establish a neural mechanism underlying treatment response. Accordingly, interpretations regarding proactive or reactive control processes remain theoretical rather than empirically confirmed^23,29^.

Third, although participants and outcome assessors remained blinded to group allocation, study coordinators who distributed devices knew participants’ assignments for logistical reasons. These coordinators did not participate in outcome assessment, data analysis, or interpretation; however, their awareness may have introduced subtle performance- or expectancy-related influences. Although we minimized such risks through a visually matched sham condition and clear separation of roles, we cannot fully exclude the possibility of residual bias. Readers should therefore interpret the magnitude of the observed treatment effects with this design feature in mind.

Fourth, we recruited participants from specialized university-based clinics, which may limit the generalizability to broader community or primary care settings. The relatively homogeneous sample and structured clinical context may not fully reflect the heterogeneity typically observed in real-world ADHD populations^43^. Future studies should replicate these findings in more diverse demographic and clinical settings to strengthen external validity^42^. In addition, researchers should also conduct implementation studies to evaluate long-term engagement^44,45^, engagement treatment adherence^43^, effectiveness across diverse subgroups (e.g., adolescents or those with comorbid conditions), and cost-effectiveness to inform real-world clinical integration and health policy decisions^46^.

STAR RUCKUS, a multitasking-based DTx, improved clinician-rated ADHD symptoms and selected cognitive measures over a 4-week period. These findings suggest potential clinical relevance within multimodal ADHD care. As a non-pharmacological digital intervention, STAR RUCKUS may provide benefit for children who experience challenges with medication adherence or tolerability. This digital intervention may serve as a complementary option within individualized treatment approaches. Future research should examine long-term durability, clarify potential neurocognitive mechanisms, evaluate effectiveness across diverse populations, and assess the health–economic implications of integrating digital therapeutics into ADHD care.

## Methods

### Study design

This study was a multicenter, stratified (by sex and medication status), randomized, single-blind, sham-controlled, parallel-group trial conducted at two university hospitals in Korea. We recruited 122 children aged 6–12 years diagnosed with ADHD according to the DSM-5 or ICD-10 criteria between April 15, 2024, and March 6, 2025. All patients were screened for eligibility prior to enrollment.

### Ethics approval and trial registration

This study was conducted in accordance with the principles of the Declaration of Helsinki. The study protocol was approved by the Institutional Review Board (IRB) at each participating institution (Seoul National University Hospital, approval no. D-2311-112-1485; Hanyang University Hospital, approval no. HYUH 2023-11-029-006). Written informed consent was obtained from the parents or legal guardians of all participating children prior to enrollment. The trial was registered at ClinicalTrials.gov (NCT07173439) on September 15, 2025.

### Participants

Participants were children aged 6–12 years diagnosed with ADHD according to DSM-5 or ICD-10 criteria. We screened 156 candidates, of which 122 met the eligibility requirements. Key inclusion criteria included clinical stability, IQ ≥ 80, and a minimum severity threshold on the Korean ADHD Rating Scale (K-ARS), a validated Korean adaptation of the ADHD Rating Scale, defined as a total score of at least 22 for boys and 18 for girls.

We excluded children with major psychiatric comorbidities, significant sensory or medical impairments, recent initiation of behavioral interventions, and intellectual functioning below the average range (IQ < 80). Supplementary Table 1 provides the full eligibility criteria.

### Randomization and masking

We randomly assigned eligible participants in a 1:1 ratio to the active intervention or control group stratified by sex (male/female) and medication status at screening. We defined medication status strata as follows: (1) a medicated group, comprising children whose ADHD medication regimen (dose and frequency) had remained stable without changes for at least one month prior to baseline, and (2) a non-medicated group, consisting of children who had not used methylphenidate or atomoxetine-based medications or those who agreed to undergo a 14-day washout period before the baseline assessment. For psychiatric medications not directly related to ADHD symptoms, the clinical investigator made determinations regarding continuation or discontinuation on an individual basis.

A statistician not involved in the study independently generated the randomization scheme using validated SAS software. We ensured that outcome assessors remained strictly blinded to intervention allocation throughout the study to preserve objectivity and minimize assessment bias. However, logistical constraints prevented the study coordinators responsible for practical procedures—such as enrolling participants, obtaining separate informed consent from children and their legal guardians, and distributing intervention devices—from being blinded to group allocation. To minimize bias, these coordinators did not participate in collecting, analyzing or interpreting outcome measures.

### Intervention

STAR RUCKUS is a DTx designed to enhance cognitive control in children with ADHD through personalized adaptive multitasking training. The intervention simultaneously combined two tasks that collaboratively engage proactive cognitive control mechanisms, including sustained working memory maintenance and inhibitory processes. The intervention involved two simultaneous tasks: (1) a visuomotor driving simulation requiring participants to tilt the tablet to dodge obstacles and collect rewards, and (2) an adaptive N-back task, in which participants continuously identified whether each stimulus matched the one presented previously. An adaptive algorithm dynamically adjusted task difficulty based on individual performance.

Participants completed five daily sessions (30–40 min) for at least 5 days per week over 4 weeks, aiming for a cumulative total of 100 sessions. Participants completed each session in a quiet, distraction-free environment with appropriate audio levels and maintained a comfortable posture. They set the tablets to “Do Not Disturb” mode during sessions to prevent distractions from notifications, calls, or messages.

We systematically monitored adherence and adverse events through weekly structured telephone calls or remote online surveys throughout the intervention period. We assessed adherence by extracting anonymized log data from the application and recording whether participants initiated sessions and how many they completed. We documented all adverse events in accordance with International Conference on Harmonization Good Clinical Practice (ICH-GCP) guidelines. In addition, we provided an emergency contact number in case of adverse events. The application included gamification elements and in-app rewards, allowing children to build customized villages, spaceships, and characters using resources earned through training to sustain motivation and engagement.

The study delivered interventions through an iPad Mini 6 tablet (Apple Inc., USA), with the active digital therapeutic or sham-control application pre-installed. The sham-control application provided a visuomotor driving simulation identical to the active intervention in terms of interface, engagement, session duration, and expectancy. However, we deliberately excluded the adaptive N-back cognitive training component, resulting in a driving-only application.

### Procedures

Potential participants underwent screening from Day 7 to Day 0, during which we obtained written informed consent, assigned screening numbers, and completed eligibility assessments. The screening assessments included clinical evaluations using the K-ARS, clinical interviews, intelligence assessments, medical history, neurological examinations, and physical examinations to determine eligibility.

Eligible participants returned on treatment initiation day (Day 0), were randomly assigned to the active intervention or sham-control group and received their designated digital therapeutic device (iPad Mini 6, Apple, USA). Intervention delivery followed a standardized protocol to ensure consistency across participants.

Within 5 days of completing the intervention, participants returned for follow-up assessments, during which they repeated the symptom severity and cognitive function evaluations conducted at screening. Outcome assessors, who remained blind to participants’ group allocation throughout the study, conducted follow-up assessments. Visual analog scales (0–10 points) quantified user feedback, including overall satisfaction and perceived difficulty of the intervention. The participants also provided qualitative feedback on potential improvements through open-ended questions. We securely stored all study data in encrypted databases and used anonymized participant codes throughout data handling and analysis.

### Outcome measures

The primary outcome was the mean change in clinician-rated ADHD symptom severity from baseline (Day 0) to post-intervention (Week 4), assessed using the clinician-administered K-ARS. The K-ARS is a validated Korean adaptation of the ADHD Rating Scale standardized in a prior validation study^47,48^. It comprises 18 items rated on a four-point Likert scale (0–3), with total scores ranging from 0 to 54. Higher scores indicate greater symptom severity.

The K-ARS includes two subscales assessing core ADHD domains: inattention and hyperactivity–impulsivity. Summing the nine relevant items provides the subscale value, with higher scores indicating greater severity. Considering that the intervention primarily targets EF, which is a common impairment in ADHD, we selected clinician-rated ADHD symptom severity as the primary clinical outcome, reflecting the functional impact of the intervention. Board-certified psychiatrists, blinded to the group allocation throughout the study, conducted the primary clinical assessments.

Secondary outcome measures included neuropsychological, behavioral, medication-related, and patient-centered endpoints, assessed at baseline and within five days following intervention completion (Week 4). Professionally trained clinical psychologists, blinded to intervention allocation, conducted neuropsychological assessments, including performance on the ADHD Diagnostic System (ADS), Stroop Color-Word Test (SCWT), and Children’s Color Trails Test (CCTT).

The ADS is a validated computerized continuous performance test standardized for Korean children that measures sustained attention and inhibitory control using nonverbal visual stimuli^49^. The system comprises one target stimulus (square containing a triangle) and two non-target stimuli (squares containing a square or a circle), each presented individually for 100 ms every 2 s. Participants pressed for a response key for targets and withheld responses for non-target stimuli. The outcome metrics included omission errors (inattention), commission errors (impulsivity), and response time variability (sustained attention), which we presented as age-adjusted T-scores based on normative data. Lower T-scores indicated better performance. We reported the visual sensitivity index (d′; perceptual sensitivity distinguishing targets from non-targets), mean response time, and visual response bias (β; response tendency) as raw scores.

The SCWT is a validated Korean adaptation^50^ of the original Stroop test developed by Golden^51^. It assesses cognitive control, selective attention, response inhibition, and interference control. The test comprises three 45-second subtests: word, color, and color–word interference. In the word subtest, participants rapidly read aloud words printed in black ink. In the color subtest, participants quickly named the colors presented as solid patches. Finally, in the color–word interference subtest, participants named the ink color of incongruently printed color words by suppressing automatic word-reading responses. This subtest specifically assessed sustained attention and inhibitory control by requiring selective responses to target color stimuli despite interference.

The CCTT is an adaptation of the Trail Making Test, specifically developed for Korean children^52^. CCTT comprises two conditions: CCTT-1 and CCTT-2. The participants completed each condition as quickly and accurately as possible. In CCTT-1, participants sequentially connected numbered circles (1 → 23) to assess their psychomotor speed and sequential processing. In CCTT-2, participants alternated between two colors while sequentially connecting numbers (e.g., pink 1→yellow 2→pink 3), which measured divided and sustained visual attention. Errors were corrected immediately. Outcome measures included completion times (seconds) for CCTT-1 and CCTT-2, and the Interference Index B, calculated as the difference between CCTT-2 and CCTT-1 completion times. Scores were presented as age-adjusted T-scores, with higher scores indicating better cognitive performance.

In addition to neuropsychological outcomes, secondary endpoints encompassed behavioral, medication-related, and patient-centered measures. Behavioral outcomes included the Clinical Global Impression–Improvement (CGI-I)^53^, a clinician-rated, seven-point scale evaluating global improvement or worsening of ADHD symptoms relative to baseline. Medication-related outcomes compared changes in ADHD medication dosage between the active intervention and sham-control groups from baseline to Week 4. Patient-centered outcomes included overall satisfaction and perceived intervention difficulty of the intervention, measured at Week 4 using visual analog scales (0–10 points). Participants also provided qualitative feedback on potential improvements and indicated their willingness to pay for the intervention.

### Statistical Analysis

Based on prior data from a randomized controlled trial of children with ADHD^24^, we calculated the required sample size to ensure adequate power (80%) at a two-sided significance level of 5%. Given a potential dropout rate of 30%, we set the final target sample size at 120 participants, with 60 per group.

We conducted all statistical analyses according to a prespecified statistical analysis plan. Independent statistical personnel, blinded to the group allocation throughout the study, performed the analyses to minimize potential bias. We included all randomly assigned participants who met the minimum usage criteria (i.e., at least one logged intervention session) in the primary analyses, using a modified intention-to-treat approach (full analysis set). Specifically, we conducted the primary and secondary efficacy analyses using the full analysis set, defined as all randomized participants who logged into the application at least once and provided at least one post-baseline primary outcome measurement.

We handled missing primary and secondary outcome data at post-intervention (Week 4) using the last observation carried forward (LOCF) method. We did not perform additional imputation for other missing variables and analyzed all available data. We summarized safety data descriptively for all participants who received the intervention at least once (Safety Set, SS). We prospectively recorded and summarized adverse events in accordance with ICH-GCP guidelines.

We analyzed the primary outcome—the mean change in clinician-rated ADHD symptom severity measured by the clinician-administered K-ARS—using analysis of covariance (ANCOVA). We adjusted for baseline K-ARS score, sex, and medication status as covariates. We reported between-group differences in adjusted mean changes with exact *p*-values, and we set statistical significance at a two-tailed α of 0.05. For ANCOVA models, we quantified effect size using partial eta-squared (*ηp*²).

We analyzed secondary outcomes similarly, using ANCOVA with the same covariates as in the primary outcome analysis. Depending on the data distribution, we used paired t-tests or Wilcoxon signed-rank tests to analyze within-group changes in secondary outcomes. To address potential Type I error inflation from multiple comparisons across the 12 neurocognitive outcomes, we applied the Benjamini–Hochberg false discovery rate (FDR) correction with a threshold of 0.05, reporting both unadjusted and adjusted *p*-values. We analyzed the CGI-I separately as a prespecified secondary endpoint because it represents a clinician-rated global assessment distinct from the cognitive tasks.

We also assessed within-group changes from baseline to post-intervention using paired t-tests and Wilcoxon signed-rank tests to examine the magnitude and significance of changes within each treatment group separately. We further strengthened the robustness of primary results by conducting additional robust regression analyses to examine the influence of outliers and departures from normality.

In addition, we conducted responder analyses for K-ARS outcomes. Responders were defined as participants achieving a ≥30% reduction from baseline to post-intervention^54^. Between-group differences in responder rates were tested using chi-square tests (or Fisher’s exact tests when expected cell counts were <5), and effect sizes were summarized using Cramer’s V.

Finally, we performed exploratory post hoc analyses (e.g., subgroup analyses stratified by medication status and additional adherence- or age-related filters described in the Results). These analyses were considered hypothesis-generating and were interpreted cautiously; no formal adjustment for multiplicity was applied. For exploratory between-group comparisons, we used ANCOVA with the same covariates as in the primary analysis when model assumptions were considered reasonable; otherwise, we used the Mann–Whitney U test. For exploratory effect sizes, we reported Cohen’s d for continuous outcomes and rank-biserial correlation for nonparametric comparisons.

We planned no interim analyses and conducted none during the study period. We performed all statistical analyses using R software (version 4.5.1; R Foundation for Statistical Computing, Vienna, Austria), employing the “car” (ANCOVA), “emmeans” (post hoc comparisons), “effectsize” (effect sizes), and “zoo” (LOCF imputation) packages.

## Supplementary information

Supplementary information

## Data Availability

The datasets generated and/or analyzed during the current study are not publicly available due to ethical restrictions and patient confidentiality agreements, but de-identified data are available from the corresponding author (J.M.) upon reasonable request, subject to institutional and national ethical guidelines and appropriate institutional approvals.

## Code availability

The code used for statistical analyses is available from the corresponding author upon reasonable request.
