# Supplementary material for: Multicenter randomized trial of a digital therapeutic game for executive function in children with ADHD
Source: NPJ Digit Med. 2026 May 27;9:645. doi: 10.1038/s41746-026-02721-3 (PMC13493770; doi:10.1038/s41746-026-02721-3)
Supplement: Supplementary file 1 [file 41746_2026_2721_MOESM1_ESM.pdf]

Supplementary Information

Supplementary Table 1. Full eligibility criteria

Inclusion Criteria

Eligibility criteria included the following:

- Children and adolescents aged ≥6 and <13 years at the time of informed consent
- Diagnosed with Attention-Deficit/Hyperactivity Disorder (ADHD) according to DSM-5 criteria or ICD-10 code F90.0, as confirmed by a qualified clinician
- A score of ≥22 for boys or ≥18 for girls on the Korean ADHD Rating Scale (K-ARS) at screening
- No changes in medication dose or regimen within 1 month prior to screening
  • Participants may be medication-naïve or currently receiving methylphenidate or amphetamine-based treatment
  • If currently treated, they must undergo a 14-day washout period prior to enrollment if willing to participate
- Considered medically healthy based on medical history and vital signs collected at screening
- Able to use a smartphone or tablet device that meets the study's minimum technical requirements without difficulty
- The participant and their legal guardian voluntarily agree to participate, and they provide written informed consent

Exclusion Criteria

We excluded participants meeting any of the following criteria:

- Presence of any of the following psychiatric comorbidities at baseline, as diagnosed by a qualified psychiatrist:
  - Chronic tic disorders requiring medication
  - Tourette's disorder
  - Major obsessive-compulsive disorder
  - Schizophrenia
  - Bipolar disorder
  - Post-traumatic stress disorder (PTSD)
  - Conduct disorder
  - Other childhood-onset psychotic disorders
  - (Note: mood disorders are not considered exclusionary)
- An intelligence quotient (IQ) below 80, as assessed by the Wechsler Intelligence Scale for Children (WISC)
- Presence of congenital genetic disorders

- Uncontrolled or hospitalization-requiring systemic medical conditions at baseline
- History of suicide attempt within the past 3 months, or currently deemed high-risk for suicide by a psychiatrist
- Initiation of or ongoing cognitive behavioral therapy (CBT) within the past 3 months
  • Includes any form of CBT, regardless of target condition (including ADHD)
- Presence of significant sensory impairments (e.g., vision or hearing) or cognitive impairments that may interfere with participation

Any other condition which, in the investigator’s opinion, may render the participant unsuitable for the study

Supplementary Table 2. Within- and Between-Group Changes in Secondary Neurocognitive Outcomes (FDR-adjusted)

	STAR RUCKUS (N=56)				Sham Control (N=58)				Between-group		
	Pre (M, SD)	Post (M, SD)	p	d	Pre (M, SD)	Post (M, SD)	P	d	P	P (FDR-adjusted)	ηp²
ADS											
Visual Omission Errors	68.91 (19.96)	72.86 (21.66)	.126 w	0.21	70.36 (20.76)	77.52 (20.65)	.007 t	0.36	.436	.523	0.00
Visual Commission Errors	72.45 (18.88)	61.27 (19.35)	<.001 t	-0.60	76.19 (21.59)	67.95 (21.45)	<.001 w	-0.46	.356	.475	0.00
Visual Mean Reaction Time	61.55 (12.45)	65.41 (15.13)	.010 t	0.31	58.81 (16.31)	62.36 (19.62)	.008 w	0.20	.924	.924	0.00
Visual Reaction Time Standard Deviation	68.41 (18.61)	67.38 (16.84)	.691 t	-0.13	69.79 (19.63)	72.66 (19.55)	.181 w	0.13	.298	.511	0.02
Visual Sensitivity Index (d′)	2.71 (0.97)	2.80 (0.91)	.430 t	0.28	2.35 (1.01)	2.26 (0.96)	.560 w	-0.15	**.021**	.252	0.04
Visual Response Bias (β)	0.67 (0.49)	1.20 (2.16)	<.001 t	0.44	0.86 (1.00)	0.30 (1.31)	<.001 w	0.28	.066	.264	0.02
Stroop											
Color Word Condition	44.91 (10.83)	53.20 (13.18)	<.001 t	0.73	45.43 (12.58)	49.24 (13.78)	.003 t	0.40	**.025**	.150	0.04
Color Condition	45.80 (11.77)	50.70 (12.60)	<.001 t	0.50	46.29 (11.96)	48.95 (11.13)	<.001 w	0.39	.124	.372	0.02
Word Condition	44.09 (9.94)	48.95 (11.03)	<.001 w	0.56	43.62 (11.66)	46.48 (12.31)	.004 t	0.38	.147	.294	0.02
CCTT											
CCTT-1 completion time	50.96 (10.68)	52.29 (10.67)	.369 t	0.12	50.59 (10.95)	51.17 (12.05)	.685 t	0.12	.791	.864	0.00
CCTT-2 completion time	50.54 (10.49)	55.43 (10.28)	<.001 t	0.43	50.26 (10.13)	53.93 (8.25)	.004 w	0.43	.309	.464	0.00
Interference Index B	49.52 (10.24)	54.16 (11.29)	.001 w	-0.34	49.72 (10.51)	53.33 (6.92)	.038 w	-0.27	.142	.341	0.01

Note. ADS = ADHD Diagnostic System; SCWT = Stroop Color–Word Test; CCTT = Children's Color Trails Test. Values are presented as mean (SD). Superscripts denote statistical tests: ^{t} two-sample t test; ^{w} Wilcoxon rank-sum test; ^{pt} paired t test; ^{pw} Wilcoxon signed-rank test. Within-group P values were derived from paired comparisons. Between-group P values were obtained using analysis of covariance (ANCOVA), adjusting for baseline values, sex, and medication status as covariates. P values in the between-group column are false discovery rate (FDR)-adjusted for multiple comparisons. *d* indicates Cohen's d. ηp^2 indicates partial eta squared

CONSORT 2010 checklist of information to include when reporting a randomised trial*

Section/Topic	Item No	Checklist item	Reported on page No
**Title and abstract**			
	1a	Identification as a randomised trial in the title	1
	1b	Structured summary of trial design, methods, results, and conclusions (for specific guidance see CONSORT for abstracts)	4
**Introduction**			
Background and objectives	2a	Scientific background and explanation of rationale	5-7
	2b	Specific objectives or hypotheses	7
**Methods**			
Trial design	3a	Description of trial design (such as parallel, factorial) including allocation ratio	13
	3b	Important changes to methods after trial commencement (such as eligibility criteria), with reasons	Not applicable
Participants	4a	Eligibility criteria for participants	13-14
	4b	Settings and locations where the data were collected	13
Interventions	5	The interventions for each group with sufficient details to allow replication, including how and when they were actually administered	14-15
Outcomes	6a	Completely defined pre-specified primary and secondary outcome measures, including how and when they were assessed	16-19
	6b	Any changes to trial outcomes after the trial commenced, with reasons	Not applicable
Sample size	7a	How sample size was determined	19
	7b	When applicable, explanation of any interim analyses and stopping guidelines	Not applicable
Randomisation:			
Sequence generation	8a	Method used to generate the random allocation sequence	14
	8b	Type of randomisation; details of any restriction (such as blocking and block size)	14
Allocation concealment mechanism	9	Mechanism used to implement the random allocation sequence (such as sequentially numbered containers), describing any steps taken to conceal the sequence until interventions were assigned	14
Implementation	10	Who generated the random allocation sequence, who enrolled participants, and who assigned participants to interventions	14
Blinding	11a	If done, who was blinded after assignment to interventions (for example, participants, care providers, those	14-15

		assessing outcomes) and how	
	11b	If relevant, description of the similarity of interventions	15
Statistical methods	12a	Statistical methods used to compare groups for primary and secondary outcomes	19-20
	12b	Methods for additional analyses, such as subgroup analyses and adjusted analyses	20
**Results**			
Participant flow (a diagram is strongly recommended)	13a	For each group, the numbers of participants who were randomly assigned, received intended treatment, and were analysed for the primary outcome	6
	13b	For each group, losses and exclusions after randomisation, together with reasons	6, 31
Recruitment	14a	Dates defining the periods of recruitment and follow-up	6
	14b	Why the trial ended or was stopped	6
Baseline data	15	A table showing baseline demographic and clinical characteristics for each group	6, 30
Numbers analysed	16	For each group, number of participants (denominator) included in each analysis and whether the analysis was by original assigned groups	6
Outcomes and estimation	17a	For each primary and secondary outcome, results for each group, and the estimated effect size and its precision (such as 95% confidence interval)	7-8, 30
	17b	For binary outcomes, presentation of both absolute and relative effect sizes is recommended	7
Ancillary analyses	18	Results of any other analyses performed, including subgroup analyses and adjusted analyses, distinguishing pre-specified from exploratory	8
Harms	19	All important harms or unintended effects in each group (for specific guidance see CONSORT for harms)	9
**Discussion**			
Limitations	20	Trial limitations, addressing sources of potential bias, imprecision, and, if relevant, multiplicity of analyses	9-12
Generalisability	21	Generalisability (external validity, applicability) of the trial findings	11
Interpretation	22	Interpretation consistent with results, balancing benefits and harms, and considering other relevant evidence	11
**Other information**			
Registration	23	Registration number and name of trial registry	13
Protocol	24	Where the full trial protocol can be accessed, if available	11
Funding	25	Sources of funding and other support (such as supply of drugs), role of funders	22-23

Citation: Schulz KF, Altman DG, Moher D, for the CONSORT Group. CONSORT 2010 Statement: updated guidelines for reporting parallel group randomised trials. BMC Medicine. 2010;8:18.
© 2010 Schulz et al. This is an Open Access article distributed under the terms of the Creative Commons Attribution License (http://creativecommons.org/licenses/by/2.0), which permits unrestricted use, distribution, and reproduction in any medium, provided the original work is properly cited.

*We strongly recommend reading this statement in conjunction with the CONSORT 2010 Explanation and Elaboration for important clarifications on all the items. If relevant, we also recommend reading CONSORT extensions for cluster randomised trials, non-inferiority and equivalence trials, non-pharmacological treatments, herbal interventions, and pragmatic trials. Additional extensions are forthcoming: for those and for up-to-date references relevant to this checklist, see www.consort-statement.org.
